# Hospital Officials' Pensions

**Published:** 1922-03

**Authors:** 


### HOSPITAL OFFICIALS' PENSIONS.

REFERENCE was made in these columns last month to the position of the hospital permanent official and the absence of any scheme to provide him with a reasonable pension. The matter, however, has not been without its advocates, and King Edward's Hospital Fund for London has been courteous enough to give a considerable amount of time to the subject. A very lengthy report has been issued by a Sub-Committee of the fund appointed on April 6, 1914, and but for the war more might have been done.

The position of the permanent hospital official is very difficult, because if a pension scheme is provided it is necessary that the length of his service in the hospital world should count for pension, and not only his service with any particular institution; hence it is that the above-mentioned Sub-Committee favours a scheme on the lines of the Federated Superannuation system for Universities. This system when first adopted provided for a payment of 5 per cent. by the University and 5 per cent. by the University employee, and when the money was provided a policy of insurance or deferred annuity was taken out in an approved insurance company. This continued until the School Teachers' Superannuation Act was passed. This Act practically caused a revolt among the University people, who pointed out the inadequacy of the pension which would be secured to them as compared with the provisions of this new Act. Consequently the Universities agreed, almost unanimously, to increase their payments from 5 per cent. to 10 per cent. on the employee's salary. This increase of 50 per cent. entirely altered the position, and in many instances the University employee is in a better position than if pensioned under the Civil Service Act of 1859 *et seq.* It is understood that an application for reconsideration of the subject will be addressed to King Edward's Hospital Fund, dealing with the altered conditions in the Universities and University Schools.

It seems hardly necessary to point out the condition of the hospital employee at the present time. He is dependent upon an administration which consists of a large number of committees, varying in the view they take as to what should be the retiring allowance granted to employees, and so it comes about that when the employee is failing he may, as has frequently happened, be retired with an allowance far in excess of the Act of '59, or of such a nature that he will receive a pension more consistent with the retiring allowance of a domestic servant. Many instances of this can be quoted, but probably one of the greatest evils that beset the absence of any system is that, as the employee advances in life he clings to office when he is no longer able to perform his duties efficiently. The Board is loth to push him out, and he, not knowing what his fate may be, struggles on, to the detriment of the institution that he serves. Furthermore, the employee is dependent upon the goodwill of his committee, and when the time comes for his retirement it often happens that the members who knew him in his best days have passed away, and new members cannot know or appreciate to the same extent the services which he rendered to the charity.

It seems a little inconsistent that an institution established to care for those who need charitable help should be the last to provide for the worker when he can no longer labour. There are few callings which exact so many obligations and entail so heavy a strain on the individual as the administration of some of our great charitable institutions, and these obligations will be even greater in the future. It is, therefore, of the utmost importance to secure the very best to manage the voluntary system, but these men will not be forthcoming until there is some assurance that they will not be abandoned when their institution has had their best days.

